# Spin transport properties of n-polyacene molecules (n = 1–15) connected to Ni surface electrodes: Theoretical analysis

**DOI:** 10.1038/srep07363

**Published:** 2014-12-08

**Authors:** S. Caliskan, A. Laref

**Affiliations:** 1 Fatih University, Department of Physics, 34500, Buyukcekmece, Istanbul, Turkey; 2 Department of Physics and Astronomy, College of Science, King Saud University, Riyadh, 11451 King Saudi Arabia; 3 Department of Physics, National Taiwan University, Taipei 106, Taiwan

## Abstract

Using non-equilibrium Green function formalism in conjunction with density functional theory, we explore the spin-polarized transport characteristics of several planar n-acene molecules suspended between two semi-infinite Ni electrodes via the thiol group. We examine the spin-dependence transport on Ni-n-acenes-Ni junctions, while the number of fused benzene rings varies between 1 and 15. Intriguingly, the induced magnetic moments of small acene molecules are higher than that of longer acene rings. The augmentation of fused benzene rings affects both the magnetic and transport features, such as the transmission function and conductance owing to their coupling to the Ni surface contacts via the anchoring group. The interplay between the spin-polarized transport properties, structural configuration and molecular electronic is a fortiori essential in these attractive molecular devices. Thus, this can conduct to the engineering of the electron spin transport in atomistic and molecular junctions. These prominent molecules convincingly infer that the molecular spin valves can conduct to thriving molecular devices.

During the last decades, a striking evolution in the area of molecular spintronic devices and quantum spin transport characteristics of organic molecular junctions^1^,^2^,^3^,^4^,^5^,^6^,^7^,^8^, has gained an ample enthusiasm. The engineering and the design of electronic transport features of molecular-scale systems has received a distinguished motivation. This may yield to paramount realistic applications for the molecular electronics, more specifically diodes, wires, quantum dots and molecular transistors^1^,^2^,^3^,^4^,^5^,^6^,^7^,^8^,^9^,^10^,^11^,^12^,^13^,^14^,^15^,^16^. The engineering of spin transport in small conjugated organic molecules^9^,^10^,^11^,^12^,^13^,^14^,^15^,^16^,^17^, graphene^17^,^18^,^19^,^20^,^21^,^22^,^23^,^24^ and magnetic molecules^25^,^26^,^27^,^28^,^29^,^30^,^31^,^32^,^33^,^34^,^35^,^36^,^37^,^38^,^39^,^40^,^41^,^42^,^43^,^44^,^45^,^46^,^47^,^48^, has achieved a tremendous scrutiny because of the significant applications in the molecular spintronic devices. Interestingly, n-acenes species, the so-called, C_4n+2_H_2n+4_ belongs to the organic molecular candidates and forms one of the most fascinating materials owing to their distinctive structural geometries and fanciful electronic features. Valuable research has been established to disseminate the relationship between the crystalline and intrinsic electronic structures, and predominantly their transport characteristics. The undertaken systems, such as polyacenes which contain polycyclic aromatic hydrocarbons, constitute linear fused benzene rings. The acene molecules with shortest length, like benzene (n = 1) and naphthalene (n = 2), seem to be the most discerned aromatic compounds, however the medium molecules in solid phase, tetracene (n = 4) and pentacene (n = 5), form semiconductor compounds.

For the past few years, an exhaustive advance has been dedicated to pentacene, both in crystalline forms and thin films which are the most appropriate structures for discerning organic p-type semiconductor. Indeed, this can drive to its relevant applications for the novel opto-electronic devices, namely the organic photovoltaic cells and organic field-effect transistor (OFET)^9^,^10^,^11^,^12^,^13^,^14^. Regarding these systems, the organic light-emitting diodes, low-voltage organic thin-film transistors, and pentacene FET's represent the most suitable devices that can be utilized in diverse technological sectors. Also, it has been emphasized that the highest length of *n*-acenes series is even viable^8^,^9^,^10^,^11^,^12^,^13^,^14^,^15^,^16^ for realistic applications^14^,^15^,^16^,^17^,^18^,^19^,^20^,^21^,^22^,^23^,^24^,^25^,^26^,^27^,^28^,^29^,^30^. The longer acenes are indicated as heptacene (n = 7), octacene (n = 8), nonacene (n = 9) and decacene (n = 10). The notorious n-acene series have attained tremendous consideration by realizing experimental and theoretical studies^7^,^8^,^9^,^10^,^11^,^12^,^13^,^14^,^15^,^16^,^17^.

Several theoretical studies were undertaken to delineate the different electronic properties of these molecules. In 2006, the synthesis and stability of heptacene has been designated after long skepticism^22^, and subsequent analysis has surmised that its ground state is paramagnetic^1^,^14^. Previously, the synthesis of octacene and nonacene has been carried out by Tonshoff *et al.*^24^, and importantly the spectroscopic inspection has revealed an anti-ferromagnetic (AFM) state in nonacene^4^,^7^,^8^,^9^,^10^,^11^,^12^,^13^,^14^,^15^,^25^. It has also been stated that the shortest acene rings (with n less than six) hold a singlet state. Hence, a diradical configuration was exhibited in the open-shell singlet state^26^,^27^. Earlier theoretical investigations have illustrated for higher acene rings (more than eight) a triplet ground state with a low energy, as pointed out previously by Houk and coworkers^25^. Six acene rings possess a magnetic ground state according to the recent statement of Bendikov and collaborators^26^. Lately, Jiang et al.^29^ attained a magnetic ground configuration for longer benzene rings (more than seven). Subsequently, the shorter acenes constitute a diamagnetic state, albeit the longer acenes are envisaged to carry a magnetic spin state. It was inferred that acenes could additionally carry an increased number of unpaired electrons relatively to the number of fused rings, since polyacenes are foreseen like zigzag-edged graphene nanoribbons (ZGNR) with a larger index than two. The magnetic ground state of higher acenes could be stabilized, although the magnetism coupled with the long spin-diffusion length would produce a valuable excitement in the next generation of organic spintronic devices^27^,^28^,^29^,^30^,^31^. Accordingly, a plentiful room exists for the theoretical progress to be accomplished in this field^9^. Electrical properties of molecular devices are often addressed by measuring the transport through a molecule sandwiched between metallic electrodes^3^. The resulting transport characteristics are determined undoubtedly by both the metal-molecule contact and molecule itself. The mechanism of a molecular device is drastically controlled by the local electronic structure of numerous molecular sites. Consequently the transport features could be restricted to a few numbers of valence orbitals. A comprehensive study of the chemical bonding, as the charge transfer or hybridization, hence has a major contribution in unraveling the molecular devices. For this occasion, the metal-molecule contact should capture a valuable attention.

Despite the strides of sophisticated theoretical techniques, the electronic structures and transport characteristics of polyacenes are still under debate. We will address and focus our attention on the results of electronic structures and spin polarized electron transport of n-acenes series (with n up to 15) that are sandwiched between two Ni metallic electrodes. Our predominant consideration is to comprehend the magnetic, electronic and transport features, more precisely the results of spin polarized transmission and conductance of Ni-n-acenes-Ni junctions (n = 1–15). The molecules in concern are suspended symmetrically between 3d Ni(111) surface contacts and are aligned in both parallel (PL) and anti-parallel (APL) spin magnetic configurations through the thiol. For this purpose, density functional theory is employed in our work, and the implementation of non-equilibrium Green's function (NEGF) approach is performed for computing the electronic spin transport characteristics. Hence, our results could support the anticipated experimental studies for the transport features of finite-sized n-acene molecules with different numbers of fused benzene rings (up to 15) that are joined to Ni leads. In this line of inquiry is to inspect the effectiveness of prominent molecules as the non-magnetic spacer in a spin valve. For this reason, the acene molecules could act as spin polarizers, filters, or producing a spin valve behavior. Intriguingly, it is surmised that the smallest n-acene molecules (n = 1–5) carry almost a similar magnetic character that is controlled through the contact region between Ni electrodes and acene rings. It is revealed that the shortest acenes illustrate a slightly bigger magnetic moment than that of the higher acene rings, when they are attached to Ni electrodes. Our results show that the conductance of acenes depends on the change of the length of rings. Thus, the computed conductance with a selected acene length, such as 11 acene rings procures the largest value. This quantity could rely drastically on the strength of acene rings connected to Ni electrodes. This could expeditiously conduct to the tremendous potential applications of these systems for the molecular magneto-electronics or molecular spin valves. For this occasion, the alteration of n-acene molecules joined to Ni contacts may change the magnetic, electronic and transport features. Our theoretical results are reasonably impressive and trigger our motivation for comprehending the transport properties of these molecular-sized contacts.

## Computational method

For the spin-polarized transport calculations, self-consistent first-principles in conjunction with NEGF technique is employed. Our computational simulation is based on ATOMISTIX TOOLKIT package^49^. Generalized gradient approximation (SGGA) PBE^50^ is applied for the exchange and correlation potential. The atomic cores are replaced by nonlocal, norm-conserving scalar-relativistic Troullier-Martins pseudopotentials. For characterizing the electronic structure of valence electrons, a double ξ polarized basis sets are employed for both acene molecules and anchoring atoms, and a single ξ polarized basis set for Ni electrodes. In the case of isolated molecules, the periodic images are separated with a minimum of 10 Å which is sufficient to prevent the interaction between the images of molecules. We relax first the molecules, which yields the optimized atomic distances between the atoms in all molecular acene rings. The n-acene molecules and electrode separation is determined through the minimization of the total energy. The optimization of the two-probe systems (electrode-molecule-electrode) is performed with Quasi-Newton method. Then, the structural geometries of n-acene molecular junctions are optimized until all the residual forces on each atom are smaller than 0.05 eV/Å. A real space grid with an equivalent plane wave cutoff of 150 Ry is applied to sample the electronic density. The modeling of transport junctions are made up by attaching the molecules to Ni fcc (111) surface contact across the sulfur anchoring group. In x, y, and z directions, the k-point sampling of 3 × 3 × 100 has been chosen. In the device system, the transport is along *z* direction in which the electrodes are semi-infinite. For the electronic band structure and density matrix, we use a value of 4 × 10^−5^ through the admixture of the Hamiltonian. With this choice, we can get a better convergence criterion for the Hamiltonian. For simplicity, the Fermi level (E_F_) is in general set to be zero. T(E,V_b_) denotes the transmission coefficient at the bias voltage V_b_ and energy E, which is given by^18^: 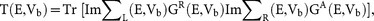
where G^A(R)^(E,V_b_) is the advanced (retarded) Green's function of the center scattering zone. Σ_L_ and Σ_R_ are the self-energies which arise from the coupling of the central scattering region to the electrodes. In the energy interval of [−eV_b_/2,+eV_b_/2], the integral of transmission coefficients accommodates the current I and [−V_b_/2,+V_b_/2] is associated with the bias window. For all the computed quantities, we select a zero bias voltage. For our prototypical systems, we utilize the architecture consisting of several n-acene molecules that are made up of fused benzene rings (the periodicity is up to n = 15) and are subsequently inserted between two metal Ni electrodes for computing the spin polarized transmission and conductance. We use anchoring groups to bind the n-acene molecules to the Ni surface through S atom linker. Thereafter, it is possible to simulate a spin-valve by attaching the molecules between two semi-infinite Ni contacts, while the magnetization directions are oriented either in PL or APL for both left and right Ni leads.

**Figure 1:**
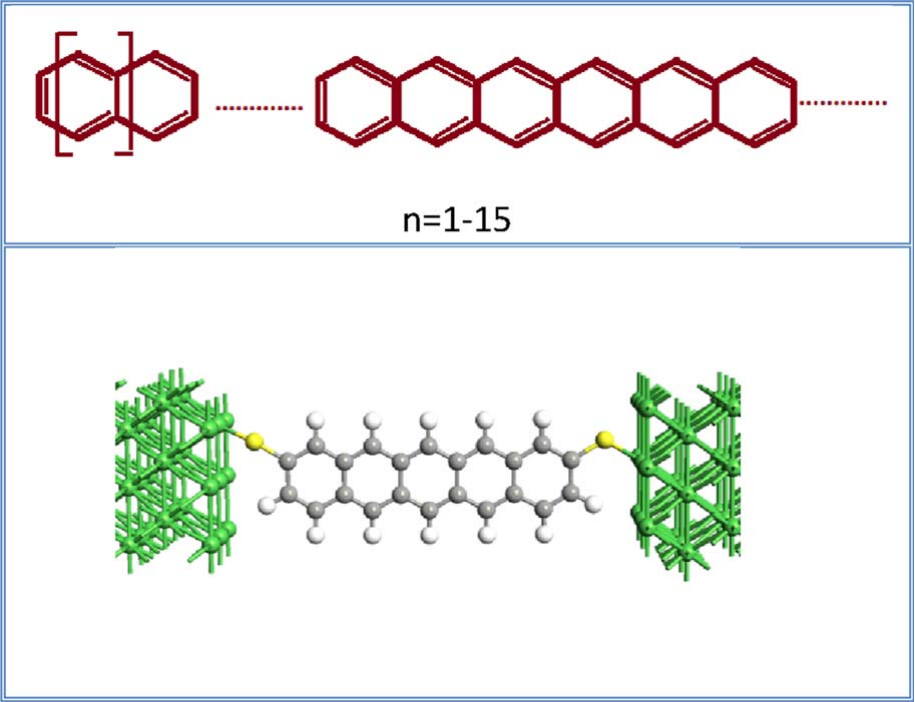
The structure model for transport properties calculations. Schematic illustration of the Ni-n-Acene-Ni junctions (n = 1–15) attached symmetrically across the S thiol. Dark grey spheres are C atoms, light dark spheres denote H atoms, yellow spheres represent S atoms and green spheres denote Ni atoms. All the optimized bond distances are indicated for both parallel (PL or FM) and anti-parallel (APL or AFM) spin configurations of the atomic Ni surface contacts.

In our theoretical analysis, the model structures of linear n-acene molecules sandwiched between Ni contacts are schematically depicted in Fig. 1. The concerned organic molecules have a conjugated π-electron system and π orbitals of C atoms and are held together by π bonding. These molecules are represented as series of benzene forming *n*-polyacenes, with a considerable different size. In our theoretical simulations, the structural model of two-probe systems that is adopted is divided into three parts from the left to the right: the left Ni electrode, the central scattering region, and the right Ni electrode. The scattering region actually includes a portion of the semi-infinite electrodes. Also, the nearest Ni atoms to the n-acenes are considered as the screening layers and the coupling interaction between acene molecules and Ni electrodes is formed across the thiol linkers (S atom). Four Ni atomic layers are chosen for the electrode cell in z-direction. To avoid the interaction between the device and its mirror images, an enough larger vacuum layer is incorporated in the electrode cell for x and y directions. The n-acene molecules with sulfur ends connected together to four atomic layers in the left and right electrodes, are chosen as central scattering region. Both the left and right S atoms are located above the hollow site of Ni electrode surface. The optimized distance between S and Ni surface in the left and right electrodes is about 2.19 Å, and C–H (C-S) distance is around 1.30 (1.72) Å.

## Results and Discussion

**Figure 2:**
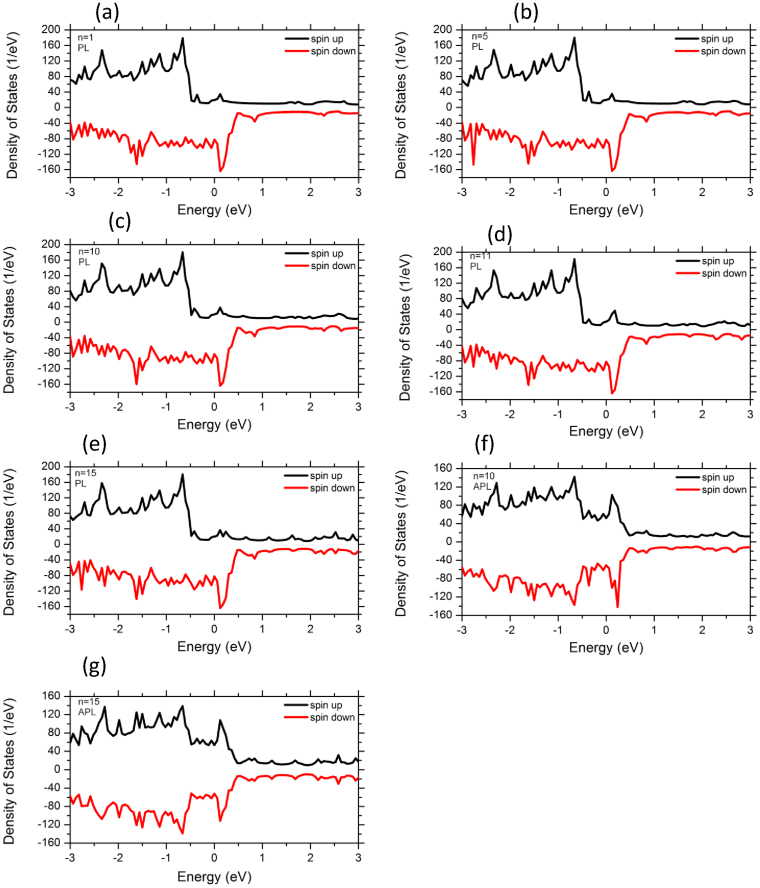
Total spin-resolved electron density (TDOS) for majority spins and minority spins of the Ni-n-acenes-Ni devices in PL spin configuration (n = 1, 5, 10, 11, 15). Note that for n = 10, 15, the TDOS are also represented for the Ni-n-acenes-Ni devices in APL spin configuration.

**Figure 3:**
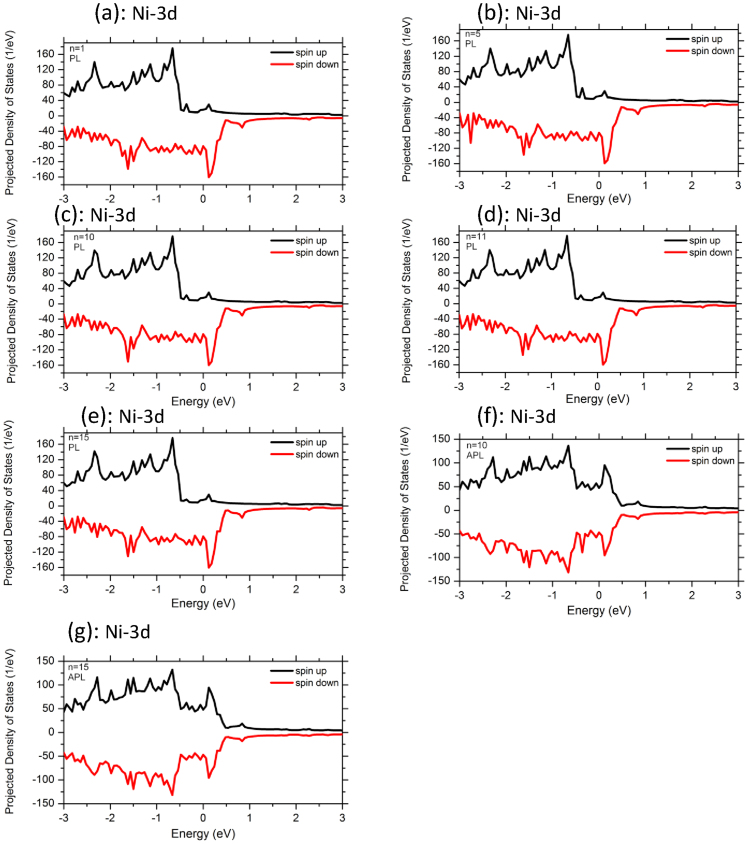
Projected density of states (PDOS) of Ni 3d states in Ni-n-acenes-Ni devices (n = 1, 5, 10, 11, 15). Note that for n = 10, 15, the PDOS of Ni 3d states are also illustrated for the Ni-n-acenes-Ni devices in APL spin configuration. Positive PDOS are for the majority spins and the negative are for the minority.

**Figure 4:**
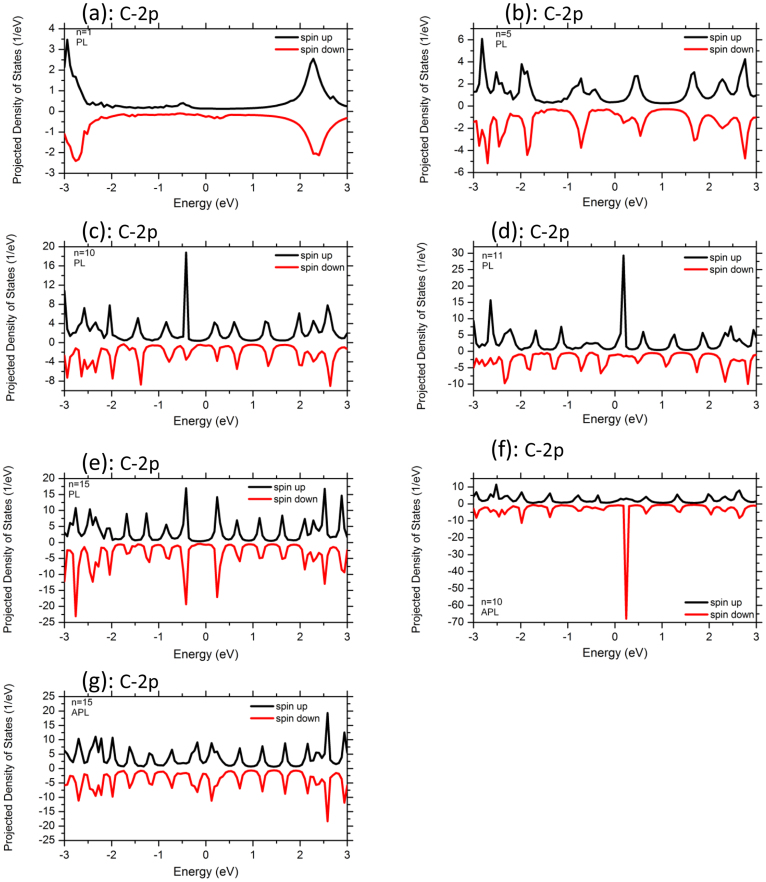
Projected density of states (PDOS) of C 2p states in Ni-n-acenes-Ni devices (n = 1, 5, 10, 11, 15). Note that for n = 10, 15, the PDOS of C 2p states are illustrated for the n-acenes suspended between Ni electrodes with magnetic moments oriented APL. Positive PDOS are for the majority spins and the negative are for the minority.

To analyze the electronic structures and transport properties of the molecular-sized junctions, we involve in our work the dependence of Ni metallic electrodes on the variation of acene rings length (n = 1–15). Here the left and right Ni electrodes are joined in PL and APL magnetic configurations. These systems can be utilized as prototypical cases of molecular spin-valves. We first discuss the results of total spin-resolved density of states (TDOS) of Ni-n-acenes-Ni devices as well as the spin polarized projected density of states (PDOS) on Ni 3d and C 2p atoms for both spin-up and spin-down carriers (see Figs. 2,3,4). For all the devices, the TDOS display a high spin polarization nearby E_F_ at 0.15 eV for both spin up and spin down channels. The topologies of TDOS slightly resemble the Ni bulk characteristics (as shown in Figs. 2(a) – (g)). At this point, the electronic states at the vicinity of E_F_ are mainly originated from the mixture of Ni 3d and C 2p characters. The PDOS of Ni 3d states exhibit similar behavior as the TDOS of devices composed of long and short acene molecules, although the number of rings varies from 1 to 15 (see Figs. 3 (a) – (g)). In the purpose to explore the lengths effect on acene rings attached to Ni surface through S atom connectors, we examine also the PDOS of C 2p states. The PDOS over all carbon atoms are illustrated at Figs. 4(a) – (g). One clearly notice that the states of short acenes (n = 1–5) are steadily spin-split and are broaden under the effect of Ni leads. It is worth noting that the spin-degeneracy of the molecular orbitals resulting from the shorter n-acenes, is lifted because of Ni lead contacts. Notably, one can still identify C 2p states at E_F_ for both spin-up and spin-down electrons (between −0.5 and 0.5 eV). This means that these acenes induce a net spin polarization over the entire molecule showing a magnetic state. The n-acene molecules that are made up of fused benzene rings, form π bonding and π* anti-bonding orbitals. These molecules possess also σ bonding and σ* anti-bonding orbitals and this is due to in-plane orbital overlap. The σ states are governed by the significant bonding-anti-bonding splitting than that of π states, since they intervene by direct orbitals overlap and are stable states. Evidently, both σ and π orbitals are filled and the anti-bonding orbitals remain unoccupied. The π and π* states of C atoms are controlled by the energy shifts which would rather drive to a spin polarized density of states near E_F_. The C-C coupling engenders a spin polarization for all the systems, since the π bonding states contribute mainly to the electrical conductance for the shortest acene rings. Conversely for higher acenes, our results demonstrate that the induced magnetic moments on C atoms slightly decrease (see Table I). A spin-splitting is significantly pronounced in PDOS of C 2p atomic orbitals for longer acenes (see Fig. 4). This is due to the frontier orbital of highest-occupied molecular orbitals (HOMO) and lowest-unoccupied molecular orbitals (LUMO) which illustrate a prominent assistance through the C 2p_π_ orbitals. It is also clearly seen that the number of peaks of C 2p states increases with the augmentation of numbers of acene rings. These states are accommodated by the coupling of n-acene molecules to the Ni surface contacts. Interestingly, the PDOS of C2p states exhibit a high-spin polarized character for both spin up and spin down states for molecules consisting of 10, 11 and 15 rings, respectively. For PL configuration (n = 11), a sharp peak is mainly present at C sites for spin-up channel around 0.2 eV and distinct structures between −0.7 eV and −0.3 eV for sin-down channel (see fig. 4) and the system illustrates a high-spin polarized behavior. For the 11 acene rings, the sharpness of PDOS peak for C 2p bands is significantly higher at the upper edge states (majority spin) than that of the lower edge ones (minority spin) (see fig. 4). This signifies a strong coupling for the upper edge state and weak coupling, i.e., the lower one is more localized. Apparently, the net spin polarization is not localized over the whole molecule, but its magnetism is local. The spin-up states above 0.2 eV are anticipated to be part of π bonding and π* anti-bonding orbitals. Eventually, the PDOS of C2p states depend on n-acene molecular sizes that are sandwiched between the Ni leads. The linearly fused benzene rings provide interesting electronic properties because of the conjugated π-electron system. For APL configuration (n = 10), the occurrence of sharp PDOS peak at 0.25 eV, is mainly located at C sites for spin-down channel. It is well noticed from the PDOS that the C 2p states has characteristic peaks at −0.2 and 0.2 eV for both spin channels (n = 15 in APL magnetic configuration). Regardless the extensive interest in the characterization of homologues higher acenes, the detailed comprehensive of electronic properties in large oligoacenes bounded with pentacene (*n* > 5) is still under scrutiny.

It is conspicuous that the experimental gap of gas phase of pentacene is about 5.54 eV^43^. Due to the coupling between the short acene molecules and Ni metallic leads, this gap will vanish. For the sake of comparison between all acene molecules, the spin-up states are positioned at the upper edge, whereas the spin-down states are located at the lower edge. This observation traces back to the hybridization between Ni and C states mediated by S character. In Ni-n-acenes-Ni junctions, the PDOS of C 2p states form sharp resonant peaks which are situated in the energy window between −0.5 and 0.5 eV (see Fig. 4) and this is valid for higher acenes. On the other hand, in the case of acene molecules varying from one to five rings, the PDOS of C 2p states illustrate a significant spin polarized. The relative contribution and numbers of resonant peaks of two spin species is an obvious consequence of the magnitude of spin splitting and position at Fermi level. Indeed, this implies that the number of resonant peaks is reduced for these spin species (n = 1–5). Hence, the C 2p PDOS are manifold and become sharper near E_F_ for long molecules, whereas the PDOS of C 2p levels are continuous and flat in the vicinity of E_F_ for shorter rings. In fact, the coupling of the smaller acene molecules to the electrodes resolves eventually magnetic states. For the entire junction (molecules plus electrodes), we detect constantly an ultimate spin configuration for all our systems. In general, the levels of electronic states are broadened and consequently a charge transfer occurs between Ni leads and HOMO of molecule. While the majority HOMO is completely occupied, the minority one may have a partial filling. Then, the total spin polarization could be different from zero. In some situations, the coupling of the organic molecules to the electrodes breaks the axial symmetry of the molecule with the induced magnetic moment. Notably, when the smaller rings are attached to the metallic surface their electronic and transport properties are influenced. At this stage, our study is predictive, since to the best of our knowledge there is no experimental evidences which report the transport properties of n-acene molecules suspended between Ni metallic leads.

We compute the magnetic moments of all polyacenes sandwiched between Ni leads (n = 1–15) (see Table 1). As apparent, the magnetic moment is reduced with the increase of the number of fused benzene rings. Interestingly the molecules develop a spin-polarized ground state for acenes consisting of different lengths ranging from 1 up to 5 rings. These shorter molecular junctions induce magnetic moments because of the influence of Ni magnetic electrodes. Note that the smallest isolated n-acene molecules (n = 1–5) illustrate non-magnetic configuration in their gas phase. However, the orbital degeneracy of these systems is lifted once the molecules are attached to Ni electrodes via the thiol S linkers, driving to a fractional magnetic moment. For the small numbers of acene rings, the atoms at the upper and lower edges of the molecules have different magnetic moment. This corresponds to a magnetic local spin arrangement (see Figs. 2,3,4) which is in stark contrast with the isolated short acene molecules. Previous studies have inferred that the isolated acene molecules with less than five benzene rings cannot hold any magnetic moment and representing paramagnetic states^7^,^8^,^9^,^10^,^11^,^12^,^13^,^14^,^15^,^16^. When the short acenes junctions are anchored to Ni leads via S links, their majority spin states are more localized. This is due to the fact that the Ni surface contacts retain a finite moment. Furthermore, a quite significant magnetic moment is induced on the smaller acene molecular junctions with less than 6 acene rings (see Table 1). Note that these n-acene molecules (n = 1–5) in contact with Ni surfaces, yield to the loss of magnetic moment from Ni atoms. This situation is in sharp contrast to the molecules possessing more than nine acene rings which illustrate a slightly lower spin-polarized ground state with respect to their isolated molecules counterpart. The average magnetic moment per atom for molecular junctions with one acene ring sandwiched between Ni electrodes is found to be 0*.*604 *μ*_B_ per atom, which is the highest value resulting from the induced total moment of Ni surfaces. The magnetic state has unpaired electrons that are piled up at the edges. This would yield partial radical characters at the edge carbon atoms, and thereby implying that acenes have one spin-up and one spin-down unpaired electrons in their ground state. One can see that the total number of unpaired spin-up electrons increases if the size of acene rings diminishes. Therefore, the resulting total magnetic moment on the molecule depends entirely on the considerable different electronic coupling to Ni leads for both two spin channels of the molecule. We refer to these entities as n-acene molecules because they are composed of “aromatic” hexagonal shaped C_6_ rings with one π-orbital electron for every C atom. For all linear acenes, a magnetic character is occurring. In this state, the spin-up and spin- down electrons occupy different sites with a ferromagnetic pattern. The acene molecules have *sp*^*2*^ hybridized carbons in which C is bonded with its neighbors by σ bonds. The spatially separated magnetizations for the spin-up and spin-down electrons diminish with the size of acene.

From the experimental point of view, a significant amount of works has been devoted to the electronic transport investigations of pentacene^19^,^20^,^21^,^22^. The corresponding material has also been used as a medium for the spin-polarized transport in composite junctions^33^ with some evidence for spin-injection^34^. For the isolated higher acenes calculations, it has been previously predicted that the edges of the molecule are spin polarized^5^,^6^,^7^,^9^,^10^,^16^. In this case there are two degenerate highest HOMOs, each of them is populated by a single electron. One of the HOMOs is localized mainly on the hydrogenated C atoms at one edge of the molecule, while the other HOMO is localized on the opposite edge. These two degenerate molecular orbitals extend in a zigzag-like symmetry along the length of the molecule. Recent calculations predicted a spin singlet ground state for the higher acenes^5^,^6^,^9^,^10^,^16^ as a result of the anti-ferromagnetic coupling between the two orbitally degenerate HOMOs. Conversely the earlier works predicted for these systems a ferromagnetic (triplet) ground state^8^,^17^,^18^. The strength of the coupling between the electrodes and central molecules can also be partly implied by the charge transfer from Ni surface to the molecules. The excess electrons on the molecules are 0.08 electrons transferred from each electrode to each C atom via S atom, indicating the coupling between C and S atoms and the surface Ni atoms of the electrodes. In this case both the majority and minority spin levels, localized on the upper and lower edges of the molecules, are coupled to the electrodes via the thiol group. This signifies that the charge transfer comes from the Ni surfaces to the molecules across the tunneling. Our theoretical findings are in consensus with the previous results obtained for the isolated higher n-acenes molecules^22^,^23^,^24^,^25^,^26^,^27^,^28^,^29^. Indeed, this situation is similar to the edge-state magnetism predicted for graphene nano-ribbons^31^,^32^,^33^,^34^.

**Figure 5:**
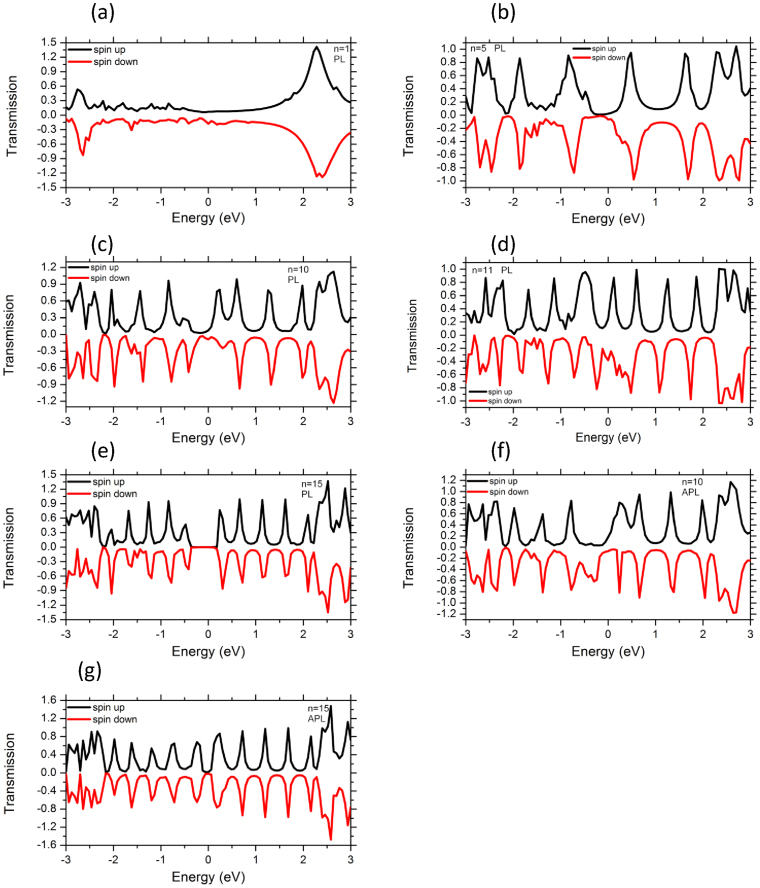
Spin-dependent transmission coefficients as a function of energy for Ni-n-acenes-Ni devices (n = 1, 5, 10, 11, 15) in PL spin configuration. Up and down indicate respectively the majority and minority spins contributions. Note that for n = 10, 15, the transmission coefficients are also given for the n-acenes suspended between the Ni leads in case of APL orientation of magnetic moments of Ni in APL spin configuration.

In order to deepen our comprehensive study regarding the transport properties of various acene rings, we present the zero-bias transmission coefficient (T(E)) results of Ni-n-acene-Ni junctions connected via anchoring groups, such as S atom. The underlying mechanism of the transmission features is associated with the wave-function overlap between the molecules and Ni metallic electrodes for all the considered configurations. Figs. 5(a) –5(g) depict the energy-dependent zero-bias electron transmission for all the representative n-acene molecule devices. In general, the features of the zero-bias transmission coefficients are different and strongly depend on the length of the molecules. For clarity, we now discuss the nature of the molecular states that are involved in the transmission at Fermi level. Overall, there is a good correspondence between the peaks of the transmission spectra and those of the DOS of all systems. Interestingly, we find a quite significant spin-polarized transmission of acene molecules, while the number of rings changes from 1 to 5. Clearly, the computed T(E) curves of magnetic molecular junctions display a qualitatively similar behavior for shorter acenes (less or equal 5 rings). The spin resolved transmission coefficient as a function of energy, is displayed in fig. 5 (a) for one ring sandwiched between Ni contacts. No sharp transport peak in T(E) is found from −0.7 to 0.8 eV for both spin channels. This effect is exclusively dramatic around E_F_, where the transport states are shrunk. Thus, this situation is a direct consequence of the re-arrangement of C 2p states (in PDOS) of acenes originating from the interaction with Ni electrodes and their hybridization in both spin orientations illustrates a reduction of the transmission near E_F_. This indicates a significant bonding between the molecule and the electrodes by showing broad resonance and a quite high conductance in the vicinity of E_F_ (at zero bias voltage). For the broadening of tunneling peaks near Fermi level, it means that T(E) changes its shape. This can be done by exerting the lower energy in the two-probe metals and therefore the HOMO is close to E_F_ which is the main transmission channel.

In n = 5, T(E) displays a broadening tunneling peak at E_F_ with reduced transport states for both majority spins and minority ones between −0.3 and 0.2 eV. At Fermi level, the transmission coefficient for majority electrons is somehow larger than that for the minority ones. The broadening of the molecular levels yields a broad transmission resonance which has a considerably large spectral weight at Fermi level and this could greatly contribute to the conductance transport. In general, a quite important spin-polarization occurs at the zero-bias transmission of shorter acenes devices, although the magnetic molecular junctions are made up of less than 6 rings (see Fig. 5). The metallic behavior is dominant in these devices. Thus, for shorter acene rings sandwiched between Ni electrodes (oriented in PL configuration), the majority spins have smaller number of resonant peaks ranging from −1 to 1 eV. Importantly, for smaller acene rings, the transmission states of the upper edge of molecule are more broadened than that of the transmission states arising from the lower edge of the molecule. The spin-splitting of the states coupled to Ni electrodes are reduced for acene devices with the variation in the number of rings from 1 to 5. Meanwhile the edges of molecules (n = 1–5) are connected to Ni leads across the thiol group, a spin magnetic moment is induced between the two edges, and therefore a spin polarized transmission.

In the case of 10 acene rings, T(E) exhibits a small HOMO–LUMO gap which is clearly visible and is well separated in the majority spin from −0.2 to 0.15 eV. Conversely, the states of the minority spin are flat and are reduced between −0.4 and 0.4 eV (see fig. 5(c)). In general, the molecular orbitals near Fermi level that are associated with different transport states are responsible for the pattern of the transmission. As a consequence, these aforementioned peaks are related to the admixture between the d states of Ni surface and p character of C atom and they represent the main reason for the magneto-resistance. For 11 acene ring devices, the narrowness of the molecular levels yields to the narrow transmission resonance which has a predominant participation to the conductance characteristic. The zero-bias transmission for majority and minority spins is different and this fact, which is supported by the analysis of the PDOS, is a simple consequence of the position of the Fermi energy and the magnitude of the spin splitting. In this regards, for n = 11, we see that the transmission for majority (spin-up) and minority (spin-down) electrons is a rather sharp function of energy near E_F_. In the minority channel, a pronounced transmission peak appears at 0*.*2 eV. Indeed, it is the resulting of the significantly high transmission peaks that are found around those corresponding energies. Fig 5(d) reveals a tunneling regime for higher energy value of 0.2 eV in the transmission coefficient. As we know, the electronic transport mechanism of the weak coupling regime is described by a sequential tunneling. That is to say when the resonant tunneling occurs, a high transmission probability appears, otherwise, the electronic transport is forbidden. Then, it is instructive to understand why the electrode-molecular interaction can induce sharper transmission peaks. For the largest length of 15 acene rings, the zero-bias transmission coefficient illustrates an energy gap between HOMO and the LUMO states which are separated from −0.5 to 0.3 eV for both spin channels. In this case, one is able to identify a corresponding HOMO and LUMO gap which yields to the quenching of the transmission for both spin-up and spin-down channels at E_F_. The results of T(E) infer that the Ni-n-acene-Ni devices (n = 15 rings) have transmission gap, which denotes a semiconducting character (fig. 5(e)). This suggests that the transport feature has a tunneling-like at Ni d – C p surface state across the thiol S. The linking of S atoms between n-acene molecules and probing electrodes play also a predominant role in tuning the transport properties of the spin polarized molecular devices.

For 10 acene rings sandwiched between Ni surface contacts oriented in APL magnetic directions, the transmission function is characterized by separated resonant peaks. T(E) shows a transmission gap at the spin up in the region from −0.5 to 0.1 eV, revealing a half-metallic character (Fig. 5(f)). In the minority spin channels, a pronounced transmission peak appears at 0*.*2 eV. Consequently, the minority spins contribute mostly to the transmission of APL configuration. At a closer look, Figs. 5(e) and 5(g) reveal that the spin-up and spin down states in the APL configuration give rise a significant contribution to the electron transport near E_F_ than those in the PL geometry. For the APL geometry with n = 15, T(E) exhibits a reduction of states from −0.15 to 0.15 eV for both spin directions. However, the transmission of this higher acene device in PL orientation is apparently suppressed from −0.3 to 0.3 V. Importantly, the number of transport peaks of T(E) increases and is manifold with the accession of the number of rings in acenes. The height of spin-polarized transmission spectra at zero bias and density of states peaks near the Fermi level of APL configuration is much larger than that of PL configuration (see Figs. 2,3,4,5). T(E) displays many tunneling peaks for the longest organic molecules, whereas fewer tunneling peaks and broadening tunneling peaks at Fermi level are obtained for shortest length of rings. In fact, the spectrum shape can be understood as follows. When the acene molecules are placed between two Ni electrodes, the coupling interaction between the molecule and two Ni electrodes will arise. So, the discrete energy levels of the molecules will be changed and shifted which is also confirmed by the charge transfer. The C 2p orbitals and Ni 3d states of surface contact contribute for the minority spin transport and give rise several resonant peaks to higher acene devices (for both PL and APL systems). For shorter acene ring devices, the transmission of majority and minority electrons shows rather a flat function of energy at E_F_. The transmission of both spin-up and spin-down electrons exhibits rather complicated behavior as a function of energy in the case of larger acene molecules (n = 15) which also participate to the electron transport. At Fermi level, the difference between the transmission of PL and APL configurations represents the measure of magneto-resistance. This demonstrates that it is viable to obtain a spin-polarized transmission of n-acenes under the effect of magnetic leads and by engineering a symmetric bonding to a metallic surface via the thiol group linkers.

**Figure 6:**
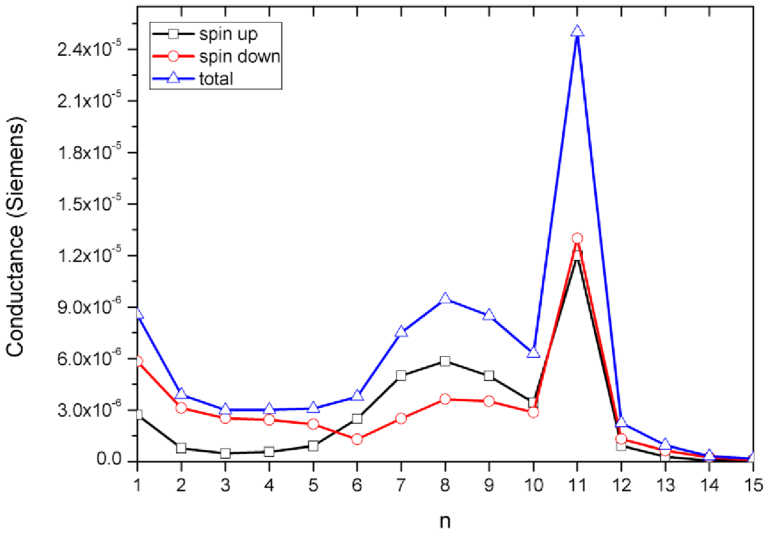
Conductance (in units of Siemens) evaluated at zero bias voltage for both spin species for n-acenes series sandwiched between Ni leads, as a function of n. The value of the conductance is sensitive to the contact geometry and the anchoring configurations.

To elucidate more clearly the origins of the above-mentioned phenomena from the electron transport properties, we analyze the spin polarized conductance for series of acene molecules coupled to Ni leads. For understanding the main source of magneto-conductance, we consider molecular junctions, where Ni contacts have influence on the size of n-acene molecular junctions. Several interesting features are apparent. In fact, to explore the reasons why the scattering region relaxation can alter the conductance, we examined the spin polarized conductance of the junction as a function of the number of rings attached to Ni-surface contact. The calculated conductance for all acenes devices with the number of rings ranging from n = 1 to n = 15 are depicted in fig. 6. For one ring sandwiched between Ni electrodes, the conductance for both spin channels is decreased and then it becomes constant for n-acenes rings spanning from 2 to 5. For short acenes, a clear π−π and σ − π bonding patterns are detected in C atoms forming a ring. It is evidently seen that the conductance of the majority spin is lower than the one of the minority spin for the shortest rings up to 5. After that we observe a spin-crossover conductance for n>5, where the spin orientation is flipped for both spin-up and spin-down channels. This is due in fact to the broadening tunneling peaks near Fermi level, it means that T(E) will change for the short acene molecules exerted between the two-probe system. This could be imperative because of the possibility of predicting (i) the coupling with the electrodes and most importantly, (ii) the position of Fermi level corresponding to the HOMO and LUMO of the molecules. These molecular orbitals would be up-shifted which indicates that the localized HOMOs states are moved near to the Fermi level of the electrodes. In fact, this could trigger a spin-flipping in the conductance. Then, it is obvious to detect a sharp peak in the conductance reaching a maximum for 11 rings. When the number of benzene rings increases up to 11, a consequent augmentation of the conductance is observed. Soon after, this highest value is reduced for 12 acene rings up to 15, and this behavior is exhibited for the conductance in both spin orientations.

The various metal/n-acenes/metal junctions (n<11) operate effectively like a tunnel barrier and causing a prompt decay in the conductance (with n > 11). This indicates that there is a strong bonding between the molecule and the electrodes producing a broad resonance and marking a higher conductance at the Fermi level for n-acenes molecule (n = 11). Most importantly, by taking a look at the transmission curve, one can see a high spin polarization at the Fermi level which yields a corresponding increase in the conductivity. The transmission is subsequently ruled by 2p and 3d orbitals of C and Ni atoms. In this respect, the π-orbitals of 11 acene molecules are highly delocalized which provide pathways to the electron transfer and consequently this can lead to higher conductance, as seen in Fig. 6. Apparently, the delocalized states participate considerably to the conductance. The trends of conductance reflect the structure of the minority spin channels and the acene indices that are involved. Conductance calculations at the zero bias show spin-valve behavior, where the Ni metallic contacts in PL spin configuration gives rise to higher conductance for n = 11. It is apparent that the resonance peak is mainly caused through the LUMO contribution. From one side, this is due to the overlapping of scattering states of the molecule to the LUMO states, or to the alignment and the shift of the molecular orbitals respective to the Fermi level of the electrode. This would drive to involving Fermi level into the LUMO state and hence a significant conductance appears. With the additional up-shift of the molecular orbitals, the conductance is reduced if EF is close to the tail of the LUMO states. The S atom contains free electrons (lone pair) which participate in the resonance of the acene chains. In general one expects that the conductance may be dominated by resonant transport either through the HOMO or the LUMO state of the molecule junctions (n = 11) sandwiched between the Ni leads. For the size of acene rings greater than 11, the spin transport reveals a tunneling-like regime and low conductance. Actually, it is interesting to mention that the nature of the conduction changes radically in the tunnel regime for the sized molecular junctions (more than 11 rings) because of the Ni contacts effects. Interestingly the dependence of the conductance on the acene length changes as n>11, i.e. as the molecules diminish the spin-polarized ground state. For the molecular length varying from12 to 15, the faster reduction of the conductance is due to the presence of σ-bonds, which can behave as tunnel barriers for the electron conduction. Thus, our calculations predict a conductance rather sensitive to the geometry of atomic junctions as well as to the type of electrodes. This is a prerequisite for the spin-valve effect, which is primarily due to spin dependent scattering. It has been observed in magnetic/nonmagnetic surface structures^51^,^52^,^53^,^54^,^55^,^56^,^57^. These results clearly show that such junctions can be used as a well controlled spin valves.

## Conclusion

Our theoretical study demonstrated the ability for tuning the spin transport properties of n-polyacenes molecules (number of fused benzene rings is up to 15). The electronic structure, magnetic, and transport characteristics are manipulated by altering the size of rings for n-acenes molecular junctions sandwiched between Ni electrodes through the S atom linker. Intriguingly, the molecules composed of smaller acene rings have a quite significant spin-polarized ground state, whereas the induced magnetic moment is diminished for the higher acenes. The stability of the molecules that are connected to the terminal Ni surfaces, occurs because of the exchange interaction in these σ − π conjugated carbon. As a matter of fact, this facilitates the strong orbital overlap between the terminal magnetic Ni atoms and non-magnetic C-atoms of small acene rings. The magnetic state of the molecular junctions is mainly due to the zigzag-shaped boundaries causing the localization of π-electrons, which form the spin orders at the edges. The length of molecular junctions reveals an increment pattern of conductance for 11 acene rings sandwiched between Ni ferromagnetic electrodes. For n = 11, the significant increment in the conductance is caused under the influence of minority spin channels, which is in turn due to the hybridization between the acene molecules and Ni contacts. Accordingly, the strength of the hybridization between the acene molecules and Ni contacts is owing to the energy position of the minority p-like states of molecule and d-like levels of Ni surface. The impact of n-acene rings on the transmission of Ni/n-acenes/Ni junctions is also elucidated. The transmission is found to be highly spin polarizedn-acene rings (n = 5–11) joined to Ni metallic electrodes. Conversely, the transmission displays a semiconducting character for 15 fused benzene rings joined to Ni metallic contacts. Consequently, the spin selectivity in the transmission depends on the effect of acene rings. As a result, the spin-dependent electron transport on the magnetic molecular junctions can be engineered by an appropriate choice of the metallic electrodes and the change of the size of n-acene rings. Based on the current technology, it is expected that the spin valve effects may be detectable for different metal-molecule junctions.

## Author Contributions

S.C. did the calculations and A.L. helped in writing the main manuscript text and all authors reviewed the manuscript.

## Figures and Tables

**Table 1:** Magnetic moments (µ_B_) and charge transfers of the Ni - n-acene - Ni junctions with various lengths, and the number of acene rings are given

Ring number	Ni atoms	S atoms	C atoms	H atoms	Total atom	Average magnetic moment per atom	Charge transfer
1	72	2	6	4	84	0.604	0.080
2	72	2	10	6	90	0.564	0.080
3	72	2	14	8	96	0.528	0.080
4	72	2	18	10	102	0.497	0.081
5	72	2	22	12	108	0.470	0.081
6	72	2	26	14	114	0.446	0.080
7	72	2	30	16	120	0.425	0.080
8	72	2	34	18	126	0.405	0.080
9	72	2	38	20	132	0.386	0.080
10	72	2	42	22	138	0.370	0.080
11	72	2	46	24	144	0.341	0.080
12	72	2	50	26	150	0.340	0.080
13	72	2	54	28	156	0.328	0.080
14	72	2	58	30	162	0.315	0.078
15	72	2	62	32	168	0.304	0.080
